# The Neighborhood Environment and Handgrip Strength: Longitudinal Findings From the Health and Retirement Study

**DOI:** 10.1093/gerona/glae242

**Published:** 2024-10-03

**Authors:** Kate A Duchowny, L Grisell Diaz-Ramirez, W John Boscardin, Rohini Perera, Scarlett Lin-Gomez, Peggy M Cawthon, Grace A Noppert, Philippa J Clarke

**Affiliations:** Institute for Social Research, University of Michigan, Ann Arbor, Michigan, USA; Division of Geriatrics, Department of Medicine UCSF School of Medicine, San Francisco, California, USA; Division of Geriatrics, Department of Medicine UCSF School of Medicine, San Francisco, California, USA; Institute for Social Research, University of Michigan, Ann Arbor, Michigan, USA; Department of Epidemiology and Biostatistics, University of California, San Francisco, California, USA; Department of Epidemiology and Biostatistics, University of California, San Francisco, California, USA; Research Institute, California Pacific Medical Center, San Francisco, California, USA; Institute for Social Research, University of Michigan, Ann Arbor, Michigan, USA; Institute for Social Research, University of Michigan, Ann Arbor, Michigan, USA

**Keywords:** Aging in place, Built environment, Neighborhoods, Older adults

## Abstract

**Background:**

Muscle strength, as measured by handgrip strength (HGS), is associated with physical function and mortality. Yet the environmental context that influences muscle strength is poorly understood. We evaluated built and social neighborhood characteristics and their association with muscle strength over time.

**Methods:**

Using data from the Health and Retirement Study (2006–2018), linear mixed models assessed how 11 built and social neighborhood variables were associated with baseline levels and changes in HGS over time.

**Results:**

Among the 20 045 respondents (mean age = 63 years, standard deviation = 9.7) with up to 4 HGS measures, 8 455 were men and 11 590 were women. Among men, residing in a neighborhood with a 10% increment higher score on neighborhood disadvantage was associated with a ~1 kg lower HGS at baseline (*B* = −0.96 kg, 95% confidence interval [CI] = −1.39 to −0.53). Similarly, each 1-point increment on the physical disorder scale was associated with a −0.39 kg lower (95% CI = −0.65 to −0.12) baseline HGS value. Among women, each 10% increment in neighborhood disadvantage was associated with a 0.29 kg lower HGS at baseline (*B* = −0.29 kg for each 10% increment, 95% CI = −0.46, −0.13). Each 1-unit increment in the number of neighborhood gyms at baseline was associated with a 0.50 kg lower HGS (*B* = −0.50, 95% CI = −0.76 to −0.23). Each 1-point increment in physical disorder was associated with a −0.12 kg lower (95% CI = −0.24 to −0.00) baseline HGS value. None of the neighborhood features were associated with the HGS rate of change.

**Conclusions:**

Findings suggest that residing in neighborhoods with greater disadvantages and physical disorders may pose challenges for HGS among middle-aged adults as they enter into older adulthood.

The majority of older adults in the United States wish to age in place (1). Aging in place is widely supported by policy-makers and clinicians as superior compared with institutional care, which is costly and associated with worse physical and mental health outcomes (2). Understanding the role neighborhood characteristics play in shaping older adults’ ability to age in their own home and community is of public health importance. This is further underscored by the fact that the neighborhood environment has been repeatedly shown to be associated with physical functioning and disability (3–5).

Older adults may be especially vulnerable to the health-promoting or health-damaging aspects of residential neighborhoods because they may be more likely to depend on local resources to maintain quality of life, are emotionally invested in their community, and spend more time at home (4). Characteristics of the neighborhood built environment, such as transportation systems, street connectivity, green space, housing density, and mixed land use, are associated with mobility, functional limitations, and disability (6–8). Older adults living in neighborhoods with a higher percentage of green space have a greater improvement in frailty status compared with those with less green space (7). Poor street conditions and heavy traffic are associated with the onset of mobility impairments both in the short and long term (5). Conditions of the neighborhood social environment are also key factors that influence health among older adults (9). For example, residents of lower socioeconomically positioned neighborhoods are at greater risk for deficits in physical functioning and disability (10). Compared with areas with high neighborhood disorder, less neighborhood disorder was found to be associated with improved physical activity levels, increased recovery from mobility limitation, and prevention of subsequent disability in older adults (11).

Despite the important role the neighborhood environment plays in shaping physical health, the role of musculoskeletal health remains an under-explored area of investigation. Muscle strength, as measured by handgrip strength (HGS), has been shown to be a primary physiologic determinant of physical disability and functional impairment (12). There are several hypothesized mechanisms linking the neighborhood environment to HGS. Specifically, it is possible that individuals who live in neighborhoods where they feel less safe may experience chronic activation of the stress response (13,14). Prior research has shown that repeated activation of the hypothalamic–pituitary–adrenal pathway may have direct consequences for muscle strength. For example, in one study that included 1200 older adults, higher cortisol levels were associated with a greater loss of grip strength over a 4-year period (15).

While prior work has examined whether physical disorder, green space, and neighborhood deprivation are associated with HGS, this work has been cross-sectional, examined a single neighborhood characteristic, or been relegated to studies with small sizes that have low external validity (7,16). Clarifying the role that the built and social neighborhood environment plays in HGS is an important area of investigation, since neighborhoods may be amenable to policies and interventions.

In this study, we evaluated whether built and social neighborhood environments are associated with HGS and to what extent these characteristics influence the trajectories of HGS over time. We leveraged linked data from the nationally representative Health and Retirement Study (HRS) to understand which built and social neighborhood characteristics may be associated with HGS. We hypothesized that neighborhoods that are more socioeconomically advantaged (ie, less neighborhood disadvantage) and with reduced physical disorder would be associated with greater baseline muscle strength and a slower rate of decline over time.

## Method

### Sample Description

We used a publicly available de-identified nationally representative cohort of 20 045 community-dwelling adults in HRS who were over the age of 50 years at baseline (2006–2018) (17). We excluded 20 776 respondents who did not have at least 1 HGS measure at age-eligible interview, had 0 weights in the 2006–2018 interview period, and had implausible BMI values of <12 or >70 and extremely low weight <24.9 kg (18). We also excluded 286 respondents with missing geographic identifiers. See the Supplementary Appendix for the sample flow diagram.

### Primary Outcome

Muscle strength was assessed via HGS, a reliable surrogate of total body muscle strength (19,20). Recently, HGS has been recognized as a biomarker of aging and has been shown to be a strong prognostic indicator of disability, morbidity, and mortality (21). This relationship is robust not only among older adults but also middle-aged and young individuals (22,23). HGS measures were comprised of readings from a Jamar dynamometer (kg) and measured every 4 years for each individual (24). We used all available measures for each respondent from 2006 to 2018 (up to 4 measures over 13 years). The maximum value from 4 trials (2 from each hand) was used based on previously validated protocols (19,25). HGS was assessed using the enhanced face-to-face sampling scheme whereby a half-random sample underwent the HGS protocol in 1 wave, and the other random half sample completed the same protocol in the following wave (24).

### Primary Exposure

We chose to include characteristics of both the built and social neighborhood environment based on the Ecological Model of Health. This theoretical framework is predicated on the idea that physical health and functioning are the result of a complex interplay of factors across multiple, nested levels, including biological/physiological factors, individual factors, and factors associated with both the built and social environment (26). Since neighborhood environment characteristics are relatively stable over time—even when people move, they often move to similar neighborhoods (27)—all neighborhood measures were assessed once based on place of residence at the time the individual entered the analytic sample (ie, first HGS assessment). Additionally, among those who were not missing on grip strength, 15.7% of respondents had moved 1 or more times. We linked census tract data from the National Neighborhood Data Archive (NaNDA), a publicly available archive of neighborhood indicator data sets (eg, street connectivity, land cover, recreation) (28). Each NaNDA data set has an 11-digit Census Federal Information Processing Series (FIPS) for linkage to HRS respondents’ FIPS code for census tract of residence. All HRS-linked census tract measures were accessed via a secure enclave following established HRS protocols and authorization.

### Built Neighborhood Environment

The built neighborhood environment was defined as those characteristics related to urban design, land-use patterns, and transportation systems (29) and included connected node ratio, an indicator of street connectivity (30); total park area (square miles) (31); number of grocery stores per 1 000 people (32); number of public transit stops (33); population density (34); average block length (30); and number of gyms/fitness centers per 1 000 people (35).

### Social Neighborhood Environment

The social neighborhood environment was captured using objective and subjective measures of social and economic factors (36). The subjective perception of the social environment—neighborhood physical disorder and social cohesion—was self-reported in the HRS using a validated set of 8 questions (37). Participants were asked to rate (on a 7-point scale) their local area (or “everywhere within a 20-minute walk”) and were asked to assess physical neighborhood disorder based on 4 indicators: presence of vandalism/graffiti, rubbish/litter, vacant/deserted homes, and crime. For the social cohesion questions, respondents were asked to assess whether they felt like they belonged, trusted individuals in their neighborhood, would receive help if needed, and whether individuals were friendly. For physical disorder, higher values reflected greater physical disorder; for social cohesion, higher scores indicated lower social cohesion/poorer community connections. Objective neighborhood socioeconomic status, socioeconomic disadvantage, was as an average of 4 census indicators (proportion of female-headed families with children, proportion of households with public assistance income or food stamps, proportion of families with income below the federal poverty level, and proportion of unemployment among the age 16+ population) ranging from 0 to 1.0 (38). We also included the number of all law enforcement organizations per 1 000 people in the census tract (39).

### Individual-Level Covariates

We included individual-level confounders in our models: age, race/ethnicity, and education. We also included hypothesized confounders/mediators: cognitive function based on the Langa–Weir classification (demented, cognitively impaired without dementia, or normal cognitive function) (40); moderate physical activity (everyday, more than once per week, once per week, 3 times per month, never); depressive symptoms as assessed by the Center for Epidemiologic Studies Depression Scale (41); a self-reported summary score of 8 medically diagnosed chronic conditions (eg, hypertension, stroke diabetes, heart disease); and body mass index (BMI). However, we note that while these variables may be considered confounders, it is possible that they may be on the causal pathway between one’s residential neighborhood environment and handgrip strength. We therefore recognize the potential for possible over-adjustment due to adjusting for mediators (42).

### Missing Data

The percentage of respondents missing at least 1 predictor was 8.5%, with BMI, social cohesion, and physical disorder accounting for the highest percentage of missing values (6.0%, 2.3%, and 2.4%, respectively). The rest of the covariates had less than 0.5% missing values. We imputed the covariates with missing values >0.5% using chained equations (see Appendix).

### Collinearity

In order to assess whether the neighborhood predictors were collinear with one another, we used the variance inflation factor (VIF). Generally, VIF values greater than 5 indicate multicollinearity may be present, and values greater than 10 indicate that multicollinearity is definitely an issue (43). Overall, VIF values were all under 5 with a mean VIF overall of 1.8, indicating low collinearity between predictors. We include the VIF table in the Supplementary Appendix.

### Statistical Approach

We fit a linear mixed effects model of HGS over time (year) and accounted for the HRS survey design. All models were run separately for men and women given known differences in HGS (44,45). We included fixed intercepts and slopes for the covariates in the model and random intercepts to account for the correlation among repeated observations for each participant over time. We specified probability weights at the respondent level and used a rescaled weight at the observation level. Details regarding the derivation of these weights, transformations, and model checks and assumptions are presented in the Supplementary Appendix. We undertook a model-building approach as follows: (1) the baseline model included individual baseline covariates; (2) Model 2 added population density (to adjust for the effect of urbanicity—more densely populated areas tend to have greater availability of resources) and neighborhood disadvantage; (3) Model 3 added built environment characteristics (street connectivity, park area, transit stops, average block length, and gyms/fitness centers) to assess the contributions of the built environment; (4) Model 4 removed the built environment characteristics and included the social environment characteristics (neighborhood disadvantage, presence of law enforcement, grocery stores, perceived physical disorder, and social cohesion) to assess the contribution of the social environment; (5) the final model, Model 5, included both the built and social environment characteristics together to assess their net contribution. Interactions between the neighborhood characteristics and time were tested to assess variation in rates of change in HGS over time. All analyses were performed with SAS/STAT 15.1, Stata 17.0, and R version 4.2.1.

## Results

### Sample Characteristics

Among the 20 045 respondents (mean age = 63 years, standard deviation [*SD*] = 9.7), 8 455 were men and 11 590 were women (Table 1). Approximately 74% of the sample identified as non-Hispanic White, 11% non-Hispanic Black, and 11% Hispanic ethnicity. Four percent of the sample identified as “other” race and/or ethnicity. Fifty-four percent of the sample attended some college or obtained a college/graduate degree. At baseline, approximately 57% of the sample reported engaging in moderate exercise once a week or more. On average, HGS was 26.2 kg for women and 43.0 kg for men. Respondents resided in census tracts with on average 2.2 square miles of open parks (*SD* = 35.9). Averaging across all census tracts, the mean score on the neighborhood disadvantage index was 10% (*SD* = 0.01 units). The average physical disorder score was 2.5 points, indicating a low/moderate amount of disorder (eg, trash on the ground, graffiti).

**Table 1.** Baseline Sample Demographics Among Men and Women Participants in the Health and Retirement Study (2006–2018, *N* = 20 045)

	Women	Men
*N*	11 590	8 455
	Weighted mean (standard error)	
Age, y	63.1 (10.6)	62.1 (8.6)
Maximum grip strength, kg	26.2 (6.8)	43.0 (9.2)
Average block length (mean length of streets in tract)	168.8 (95.0)	171.3 (86.5)
Connected node ratio	0.5 (0.1)	0.5 (0.1)
Count of transit stops reported to National Transit Map as of 2018 per census tract	7.3 (14.6)	7.3 (13.3)
Total area of open parks per census tract (square miles)	2.1 (35.8)	2.4 (35.6)
Law enforcement organizations per 1 000 people	0.3 (0.7)	0.3 (0.6)
Supermarkets/grocery stores per 1 000 people	0.6 (0.6)	0.5 (0.5)
Social cohesion index*	5.4 (1.5)	5.3 (1.2)
Physical disorder index*	2.5 (1.5)	2.5 (1.3)
Neighborhood disadvantage index	0.1 (0.1)	0.1 (0.1)
Population density as of 2010, persons per square mile	4 334.8 (11 442.3)	4 129.8 (9627.1)
Gyms/fitness centers per 1 000 people	0.3 (0.4)	0.3 (0.4)
BMI, kg/m^2^	*N* (weighted percent)	
<18.5	114 (1.0)	40 (0.4)
18.5 to <25	2 642 (25.2)	1 404 (16.3)
25 to <30	3 381 (30.5)	3 320 (41.2)
30+	4 718 (43.3)	3 230 (42.1)
Race/ethnicity		
White	7 702 (73.6)	5 907 (73.7)
Black	2 165 (11.9)	1 274 (10.6)
Hispanic	1 338 (10.2)	970 (11.1)
Other	371 (4.2)	291 (4.7)
Educational attainment		
0–12 years, no diploma	2 624 (19.4)	1 942 (19.8)
High school graduate	3 612 (28.8)	2 209 (24.8)
Some college	2 979 (27.0)	2 007 (25.5)
At least bachelor’s degree	2 374 (24.8)	2 296 (29.9)
Langa–Weir classification of cognitive function		
Normal	9 507 (84.6)	6 835 (83.9)
CIND	1 733 (12.4)	1 372 (12.9)
Demented	350 (3.1)	248 (3.2)
Frequency of moderate physical activity		
Everyday	1057 (9.2)	882 (10.5)
More than once per week	4 990 (46.1)	3 949 (48.1)
Once per week	190 (15.3)	1 492 (17.8)
Three times per month	1 288 (10.4)	839 (10.0)
Never	2 457 (19.0)	1 286 (13.6)
CES-D		
No depression (0–2)	8 822 (76.8)	7 000 (81.7)
Depressed (3+)	2 767 (23.2)	1 454 (18.3)
Chronic conditions (ever had)		
0	1 789 (18.9)	1 551 (22.8)
1	2 930 (26.5)	2 166 (28.0)
2	3 155 (25.7)	2 079 (23.0)
3–8	3 716 (28.9)	2 659 (26.1)

*Notes*: BMI = body mass index; CES-D = Center for Epidemiologic Studies—Depression Scale; CIND = cognitively impaired but not demented.

*Ratings were assessed using a 7-point Likert scale (1 = more favorable conditions; 7 = worse conditions). For physical disorder, higher values reflect greater physical disorder. Similarly, for social cohesion, higher scores indicated lower social cohesion/poorer community connections.

### Results for Women

Among women, after adjusting for individual-level covariates, HGS decreased at a rate of 0.46 kg per year (Table 2). The inclusion of neighborhood disadvantage, after adjusting for population density (Model 2), indicated a 10% higher score in neighborhood disadvantage was associated with a small but statistically significant lower HGS at baseline (*B* = −0.29 kg, 95% confidence interval [CI] = −0.46 to −0.13). On average, each additional year of age was associated with a 0.27 kg lower HGS (*B* = −0.27 kg, 95% CI = −0.28 to −0.26). When adding the built environment characteristics (Model 3), each 1-unit increase in the density of gyms at baseline was associated with a 0.50 kg lower HGS (*B* = −0.50, 95% CI = −0.76 to −0.23). The inclusion of the social environment characteristics (Model 4) revealed that each 1-point increase in the physical disorder scale was associated with a 0.12 kg lower HGS at baseline (95% CI = −0.24 to −0.00). In the final model (Model 5), which included both built and social neighborhood characteristics, neighborhood disadvantage (*B* = −0.20 kg, 95% CI = −0.39 to −0.02), density of gyms and fitness centers (*B* = −0.49 kg, 95% CI = −0.76 to −0.22), and neighborhood physical disorder (*B* = −0.13, 95% CI = −0.24 to −0.01) were associated with lower HGS at baseline. None of the neighborhood characteristics were associated with rates of change in HGS over time.

**Table 2.** Linear Mixed Model Coefficients [With 95% Confidence Interval] for Hand Grip Strength: Women. Health and Retirement Study (2006–2018, *N* = 11 590)

	Model 1	Model 2	Model 3	Model 4	Model 5
	Individual Covariates	Model 1 + Disadvantage	Model 2 + Built Environment	Model 3 + Social Environment	Model 4 + Built & Social Environment
Intercept	39.01 [36.79 to 41.23]	41.32 [39.01 to 43.63]	42.94 [38.50 to 47.38]	41.33 [38.87 to 43.78]	43.27 [38.72 to 47.82]
Neighborhood disadvantage index		−2.92 [−4.56 to −1.27]	−3.39 [−5.08 to −1.71]	−1.42 [−3.24 to 0.41]	−2.03 [−3.88 to −0.18]
Population density		−0.26 [−0.33 to −0.19]	−0.27 [−0.41 to −0.14]	−0.26 [−0.34 to −0.19]	−0.29 [−0.43 to −0.16]
Connected node ratio			−0.61 [−2.09 to 0.87]		−0.42 [−1.92 to 1.08]
Total park area			−0.00 [−0.00 to 0.00]		0.00 [−0.00 to 0.00]
Transit stops			0.00 [−0.01 to 0.02]		0.01 [−0.01 to 0.02]
Average block length			−0.20 [−0.72 to 0.32]		−0.26 [−0.78 to 0.25]
Gyms/fitness centers			−0.50 [−0.76 to −0.23]		−0.49 [−0.76 to −0.22]
Law enforcement				−0.12 [−0.32 to 0.08]	−0.12 [−0.33 to 0.09]
Supermarkets				−0.13 [−0.38 to 0.13]	−0.07 [−0.33 to 0.19]
Social cohesion				0.07 [−0.03 to 0.18]	0.08 [−0.03 to 0.19]
Physical disorder				−0.12 [−0.24 to −0.00]	−0.13 [−0.24 to −0.01]
Age (at baseline)	−0.28 [−0.29 to −0.27]	−0.27 [−0.28 to −0.26]	−0.27 [−0.28 to −0.26]	−0.27 [−0.28, −0.26]	−0.27 [−0.28 to −0.26]
Race					
White (reference)					
Black	1.17 [0.84 to 1.50]	1.43 [1.04 to 1.83]	1.43 [1.03 to 1.82]	1.45 [1.05 to 1.85]	1.44 [1.04 to 1.84]
Hispanic	−1.74 [−2.14 to −1.35]	−1.50 [−1.90 to −1.11]	−1.49 [−1.88 to −1.10]	−1.51 [−1.90 to −1.11]	−1.49 [−1.88 to −1.10]
Other	−0.98 [−1.75 to −0.20]	−1.06 [−1.82 to −0.29]	−1.06 [−1.83 to −0.29]	−1.03 [−1.78 to −0.27]	−1.03 [−1.79 to −0.27]
Education					
Less than high school (reference)					
High school	−0.09 [−0.43 to.0.25]	−0.10 [−0.45 to 0.25]	−0.10 [−0.45 to 0.25]	−0.12 [−0.47 to 0.24]	−0.12 [−0.47 to 0.24]
Some college	0.30 [−0.07 to 0.68]	0.26 [−0.11 to 0.64]	0.28 [−0.10 to 0.65]	0.23 [−0.15 to 0.61]	0.24 [−0.14 to 0.62]
College+	0.46 [0.09 to 0.82]	0.41 [0.08 to 0.74]	0.44 [0.12 to 0.76]	0.36 [0.02 to 0.70]	0.39 [0.06 to 0.72]
Cognitive function (at baseline)					
Normal (reference)					
CIND	−0.46 [−0.82 to −0.11]	−0.66 [−1.03 to −0.29]	−0.67 [−1.03 to −0.30]	−0.65 [−1.01 to −0.28]	−0.65 [−1.01 to −0.29]
Demented	−0.86 [−1.44 to −0.29]	−1.35 [−1.97 to −0.72]	−1.32 [−1.95 to −0.70]	−1.33 [−1.96 to −0.71]	−1.30 [−1.92 to −0.68]
Physical activity (at baseline)					
Everyday (reference)					
More than once per week	−0.25 [−0.68 to 0.19]	−0.25 [−0.68 to 0.18]	−0.24 [−0.67 to 0.19]	−0.24 [−0.67 to 0.19]	−0.23 [−0.66 to 0.20]
Once per week	−0.53 [−1.06 to −0.01]	−0.58 [−1.11 to −0.06]	−0.58 [−1.11 to −0.05]	−0.56 [−1.09 to −0.03]	−0.56 [−1.10 to −0.02]
Three times per month	−0.58 [−1.14 to −0.01]	−0.71 [−1.26 to −0.16]	−0.70 [−1.25 to −0.15]	−0.69 [−1.24 to −0.13]	−0.68 [−1.24 to −0.12]
Never	−1.88 [−2.36 to −1.41]	−2.09 [−2.56 to −1.61]	−2.07 [−2.55 to −1.59]	−2.05 [−2.53 to −1.58]	−2.04 [−2.52 to −1.56]
Depressive symptoms					
CES-D score 3+, at baseline	−0.89 [−1.19 to −0.59]	−0.89 [−1.19 to −0.59]	−0.89 [−1.19 to −0.59]	−0.85 [−1.15 to −0.54]	−0.84 [−1.15 to −0.54]
Chronic conditions ever had (at baseline)					
0 (reference)					
1	−0.47 [−0.91 to −0.02]	−0.45 [−0.91 to 0.01]	−0.45 [−0.91 to 0.00]	−0.44 [−0.89 to 0.02]	−0.44 [−0.89 to 0.02]
2	−1.13 [−1.49 to −0.76]	−1.12 [−1.49 to to −0.74]	−1.12 [−1.49 to −0.75]	−1.09 [−1.46 to −0.71]	−1.09 [−1.46 to −0.71]
3–8	−1.83 [−2.23 to −1.43]	−2.01 [−2.42 to −1.59]	−2.02 [−2.44 to −1.60]	−1.96 [−2.37 to −1.55]	−1.97 [−2.38 to −1.56]
BMI to (at baseline)					
<18.5 (reference)					
18.5 to <25	0.19 [0.11 to 0.27]	0.21 [0.12 to 0.30]	0.21 [0.13 to 0.30]	0.21 [0.12 to 0.30]	0.21 [0.12 to 0.30]
25 to <30	−0.02 [−0.38 to 0.34]	−0.06 [−0.44 to 0.32]	−0.06 [−0.44 to 0.31]	−0.05 [−0.43 to 0.33]	−0.05 [−0.43 to 0.33]
30+	−0.37 [−1.44 to 0.71]	−0.29 [−1.42 to 0.83]	−0.29 [−1.41 to 0.84]	−0.33 [−1.46 to 0.80]	−0.32 [−1.44 to 0.80]
Rate of change (per year)					
Time		−0.46 [−0.54 to −0.39]	−0.52 [−1.00 to −0.03]	−0.49 [−0.60 to −0.38]	−0.58 [−1.13 to −0.03]
Population density × time		0.00 [−0.01 to 0.01]	0.00 [−0.02 to 0.02]	0.01 [−0.00 to 0.02]	0.00 [−0.12 to 0.03]
Neighborhood disadvantage index × time		0.13 [−0.15 to 0.41]	0.15 [−0.12 to 0.42]	0.03 [−0.24 to 0.30]	0.08 [−0.20 to 0.35]
Connected node ratio × time			0.15 [−0.02 to 0.33]		0.14 [−0.04 to 0.33]
Total park area × time			0.00 [−0.00 to 0.00]		0.00 [−0.00 to 0.00]
Transit stops × time			−0.00 [−0.00 to −0.00]		−0.00 [−0.00 to −0.00]
Average block length × time			−0.00 [−0.08 to 0.08]		0.00 [−0.08 to 0.09]
Gyms/fitness centers × time			0.05 [0.00 to 0.09]		0.05 [0.01 to 0.10]
Law enforcement × time				0.03 [−0.02 to 0.08]	0.03 [−0.02 to 0.07]
Supermarkets × time				−0.01 [−0.05 to 0.02]	−0.02 [−0.06 to 0.02]
Social cohesion × time				−0.00 [−0.01 to 0.01]	−0.00 [−0.02 to 0.01]
Physical disorder × time				0.01 [−0.00 to 0.03]	0.01 [−0.00 to 0.03]

*Notes*: BMI = body mass index; CES-D = Center for Epidemiologic Studies—Depression Scale; CIND = cognitively impaired but not demented.

### Results for Men

After adjusting for individual-level covariates (Model 1), HGS for men declined at the rate of 0.77 kg per year (Table 3). In Model 2, after adjusting for population density, a 10% higher score on the neighborhood disadvantage index was associated with a ~1 kg lower HGS at baseline (*B* = −0.96 kg, 95% CI = −1.39 to −0.53). HGS decreased by 0.42 kg (95% CI = −0.45 to −0.40) for each year of age. We then added the built environment characteristics (Model 3) and observed no statistically significant association with HGS. Model 4 included the social environment. Each 1-point increase on the physical disorder scale was associated with a 0.39 kg lower (95% CI = −0.65 to −0.12) baseline HGS. Similarly, a greater density of supermarkets was associated with a 0.47 kg lower HGS (95% CI = −0.87 to −0.08). In the fully adjusted model (Model 5), neighborhood disadvantage (*B* = −0.68 kg, 95% CI = −1.13 to −0.22) and physical disorder (*B* = −0.40 kg, 95% CI = −0.66 to −0.13) were associated with lower HGS at baseline. None of the neighborhood characteristics were associated with rates of change in HGS over time.

**Table 3.** Linear Mixed Model Coefficients [With 95% Confidence Intervals] for Hand Grip Strength: Men. Health and Retirement Study, (2006–2018, *N* = 8 455).

	Model 1	Model 2	Model 3	Model 4	Model 5
	Individual Covariates	Model 1 + Disadvantage	Model 2 + Built Environment	Model 3 + Social Environment	Model 4 + Built & Social Environment

Intercept	49.82 [43.07 to 56.57]	52.87 [45.63 to 60.11]	50.20 [38.50 to 61.91]	53.55 [46.13 to 60.97]	51.53 [39.73 to 63.32]
Neighborhood disadvantage index		−9.61 [−13.90 to −5.32]	−10.37 [−14.65 to −6.10]	−5.73 [−10.38 to −1.08]	−6.72 [−11.28 to −2.17]
Population density		−0.25 [−0.39 to −0.11]	−0.20 [−0.46 to 0.06]	−0.24 [−0.38 to −0.09]	−0.23 [0.49 to 0.04]
Connected node ratio			1.04 [−1.33 to 3.41]		1.48 [−0.89 to 3.86]
Total park area			−0.01 [−0.02 to 0.00]		−0.01 [−0.02 to 0.00]
Transit stops			−0.00 [−0.02 to 0.02]		0.00 [−0.02 to 0.02]
Average block length			0.41 [−0.74 to 1.57]		0.28 [−0.90 to 1.46]
Gyms/fitness centers			−0.59 [−1.20 to 0.01]		−0.49 [−1.11 to 0.13]
Law enforcement				−0.16 [−0.66 to 0.33]	−0.12 [−0.61 to 0.37]
Supermarkets				−0.47 [−0.87 to −0.08]	−0.41 [−0.82 to 0.00]
Social cohesion				0.06 [−0.20 to 0.32]	0.05 [−0.21 to 0.31]
Physical disorder				−0.39 [−0.65 to −0.12]	−0.40 [−0.66 to −0.13]
Age (at baseline)	−0.43 [−0.46 to −0.41]	−0.42 [−0.45 to −0.40]	−0.42 [−0.45 to −0.40]	−0.42 [−0.45 to −0.40]	−0.42 [−0.45 to −0.40]
Race					
White (reference)					
Black	−0.80 [−1.51 to −0.09]	−0.26 [−1.01 to 0.48]	−0.28 [−1.03 to 0.47]	−0.19 [−0.93 to 0.54]	−0.21 [−0.96 to 0.53]
Hispanic	−4.07 [−4.70 to −3.44]	−3.76 [−4.52 to −3.00]	−3.80 [−4.57 to −3.02]	−3.79 [−4.54 to −3.05]	−3.82 [−4.57 to −3.06]
Other	−1.95 [−2.83 to −1.06]	−2.53 [−3.42 to −1.65]	−2.54 [−3.43 to −1.65]	−2.53 [−3.41 to −1.65]	−2.54 [−3.43 to −1.64]
Educational attainment					
Less than high school (reference)					
High school	−0.47 [−1.15 to 0.20]	−0.53 [−1.27 to 0.22]	−0.51 [−1.25 to 0.23]	−0.62 [−1.37 to 0.12]	−0.61 [−1.35 to 0.13]
Some college	0.23 [−0.38 to 0.85]	0.03 [−0.64 to 0.70]	0.06 [−0.62 to 0.74]	−0.07 [−0.76 to 0.62]	−0.05 [−0.75 to 0.65]
College+	−0.04 [−0.61 to 0.52]	−0.08 [−0.72 to 0.55]	−0.02 [−0.67 to 0.62]	−0.25 [−0.89 to 0.39]	−0.21 [−0.86 to 0.45]
Cognitive function (at baseline)					
Normal (reference)					
CIND	−1.54 [−2.20 to −0.88]	−1.64 [−2.31 to −0.97]	−1.63 [−2.30 to −0.97]	−1.60 [−2.26 to −0.95]	−1.60 [−2.25 to −0.96]
Demented	−2.57 [−3.97 to −1.17]	−3.15 [−4.57 to −1.73]	−3.12 [−4.53 to −1.72]	−3.18 [−4.62 to −1.73]	−3.16 [−4.59 to −1.73]
Physical activity (at baseline)					
Everyday (reference)					
More than once per week	−0.11 [−0.84 to 0.62]	−0.05 [−0.77 to 0.67]	−0.06 [−0.77 to 0.66]	−0.05 [−0.77 to 0.68]	−0.05 [−0.77 to 0.67]
Once per week	−0.81 [−1.60 to −0.01]	−0.90 [−1.72 to −0.08]	−0.91 [−1.72 to −0.09]	−0.87 [−1.69 to −0.05]	−0.87 [−1.69 to −0.06]
Three times per month	−0.98 [−1.91 to −0.04]	−1.12 [−2.05 to −0.18]	−1.15 [−2.08 to −0.22]	−1.13 [−2.08 to −0.18]	−1.16 [−2.10 to −0.21]
Never	−2.51 [−3.51 to −1.51]	−2.74 [−3.72 to −1.76]	−2.75 [−3.73 to −1.77]	−2.71 [−3.69 to −1.73]	−2.72 [−3.70 to −1.74]
Depressive symptoms					
CES-D score 3+ to at baseline	−1.35 [−1.92 to −0.79]	−1.34 [−1.89 to −0.79]	−1.35 [−1.90 to −0.80]	−1.22 [−1.76 to −0.68]	−1.23 [−1.77 to −0.69]
Chronic conditions ever had (at baseline)					
0 (reference)					
1	−0.90 [−1.50 to −0.30]	−0.90 [−1.50 to −0.31]	−0.91 [−1.50 to −0.31]	−0.92 [−1.50 to −0.35]	−0.93 [−1.50 to −.0.35]
2	−2.10 [−2.89 to −1.31]	−2.19 [−2.97 to −1.42]	−2.20 [−2.97 to −1.43]	−2.18 [−2.94 to −1.41]	−2.18 [−2.94 to −1.43]
3–8	−3.17 [−3.95 to −2.39]	−3.48 [−4.27 to −2.70]	−3.49 [−4.26 to −2.71]	−3.43 [−4.20 to −2.65]	−3.43 [−4.20 to −2.67]
BMI, (at baseline)					
<18.5 (reference)					
18.5–<25	0.84 [0.58 to 1.09]	0.87 [0.60 to 1.15]	0.87 [0.60 to 1.15]	0.87 [0.59 to 1.14]	0.87 [0.60 to 1.15]
25–<30	−1.53 [−2.52 to −0.54]	−1.62 [−2.66 to −0.58]	−1.63 [−2.67 to −0.59]	−1.63 [−2.67 to −0.58]	−1.64 [−2.68 to −0.60]
30+	3.09 [0.14 to 6.03]	3.28 [0.20 to 6.36]	3.30 [0.23 to 6.37]	3.32 [0.23 to 6.42]	3.35 [0.28 to 6.43]
Rate of change (per year)					
Time		−0.77 [−0.90 to −0.64]	−0.85 [−1.59 to −0.10]	−0.75 [−1.02 to −0.48]	−1.00 [−1.74 to −0.25]
Population density × time		0.01 [−0.01 to 0.03]	0.01 [−0.01 to 0.04]	0.01 [−0.01 to 0.03]	0.03 [−0.00 to 0.05]
Neighborhood disadvantage index × time		−0.09 [−0.48 to 0.30]	0.02 [−0.39 to 0.42]	−0.32 [−0.77 to 0.13]	−0.22 [−0.67 to 0.24]
Connected node ratio × time			−0.16 [−0.49 to 0.18]		−0.20 [−0.54 to 0.13]
Total park area × time			0.00 [−0.00 to 0.00]		0.00 [−0.00 to 0.00]
Transit stops × time			0.00 [−0.00 to 0.00]		0.00 [−0.00 to 0.00]
Average block length × time			0.02 [−0.10 to 0.13]		0.05 [−0.07 to 0.16]
Gyms/fitness centers × time			0.06 [−0.01 to 0.12]		0.03 [−0.04 to 0.10]
Law enforcement × time				0.05 [−0.01 to 0.11]	0.05 [−0.01 to 0.11]
Supermarkets × time				0.06 [0.00 to 0.11]	0.05 [−0.01 to 0.11]
Social cohesion × time				−0.01 [−0.04 to 0.02]	−0.10 [−0.04 to 0.02]
Physical disorder × time				−0.00 [−0.03 to 0.02]	−0.00 [−0.03 to 0.03]

*Notes*: BMI = body mass index; CES-D = Center for Epidemiologic Studies—Depression Scale; CIND = cognitively impaired but not demented.

## Discussion

In this nationally representative sample of middle-aged and older men and women, we found that the residential neighborhood environment was associated with HGS, a valid surrogate of total body muscle strength and a strong predictor of physical functioning and disability among older adults. In assessing the independent contributions of the built and social neighborhood environment, the results of this investigation yielded 3 principal findings. First, residing in neighborhoods with greater socioeconomic disadvantage was associated with significantly lower levels of baseline HGS among both men and women, even after adjusting for individual covariates and other aspects of the built and social neighborhood environment. Second, a perceived physical disorder in the neighborhood was also associated with lower HGS at baseline among both men and women. Third, when assessing individual features of the neighborhood’s social and built environment, we found that a greater density of supermarkets and gyms/fitness centers were associated with lower HGS in men and women. Our findings are consistent with our initial hypotheses that residents in more socially disadvantaged neighborhoods with more signs of physical disorder would be associated with decreased muscle strength.

Our finding that neighborhood disadvantage, an indicator of neighborhood socioeconomic position (46), was associated with lower HGS for both men and women is consistent with other research observing a strong social gradient across levels of HGS. For example, in one study that examined whether social inequities were associated with poorer physical function, falls, and HGS among 3 225 men and women aged 59–73 in the Hertfordshire Cohort Study, the authors found not owner-occupying one’s home was associated with lower HGS in men and women and poorer physical functioning in men (16). In 3 studies that examined the association between socioeconomic position (SEP) and HGS across the life course, lower SEP was associated with weaker HGS, and this association was stronger in women than in men (47–49). For example, Carney et al. found that disadvantaged SEP was associated with lower HGS for women and that the age and level of peak HGS occurred at younger ages (48). Similarly, Yusef et al. specifically found that mother’s education and father’s occupation had the strongest associations with HGS for women and that these associations were robust to adjustments for body size and childhood and adult factors (49). In a scoping review that examined the relation between neighborhood characteristics and physical frailty, 12 out of the 13 studies found that greater neighborhood disadvantage was strongly associated with an increased risk for frailty, a multifactorial condition which includes muscle weakness as a defining indicator (50).

There are several reasons why neighborhood disadvantage may be associated with lower HGS. First, residing in disadvantaged neighborhoods over the life course may affect HGS by greater exposure to toxic environments (eg, polluting sites, highways), which have been shown to be associated with poor physical functioning and reduced HGS (51,52). Second, disadvantaged neighborhoods tend to have negative peer influences on health behaviors (eg, diet, substance use, exercise), which have important implications for lifelong muscle health (53–55). Lastly, increased (and prolonged) stress exposure in disadvantaged neighborhoods can lead to inflammation and other physiological changes in the body’s immune system, which has been associated with poor muscle health (53–55).

Greater perceived neighborhood physical disorder was associated with lower HGS among women and men and may serve as a potential mechanism linking neighborhood disadvantage to HGS. Consistent with prior studies and our hypothesis, middle-aged and older adults were more likely to have lower muscle strength when living in neighborhoods with high amounts of vandalism/graffiti, litter, and vacant/deserted houses. Potential reasons for this finding may be reduced physical activity and perceptions of feeling unsafe in one’s neighborhood (56). We found that women residing in neighborhoods with a greater density of gyms/fitness centers and supermarkets had lower HGS. One potential reason for this finding may be due to the fact that neighborhoods that have a high density of supermarkets and gyms also have a high density of other amenities—that is, fast food, restaurants, bars, liquor stores, convenience stores, etc. It is possible that the inverse association could be due to these other unmeasured features that co-occur in these environments. Additionally, it is important to note that while some individuals may reside in an environment with gyms, parks, and supermarkets, it does not necessarily mean they are utilizing these amenities (56,57).

We note that the main associations observed were among the neighborhood characteristics and baseline intercept estimates of HGS. In contrast, no associations were observed among the slope estimates. It is possible that trajectories of HGS are established earlier in the life course, and by age 51+ the neighborhood context does little to alter this trajectory. Prior studies have shown that midlife is a critical window for the preservation of HGS, where a divergence in trajectories of physical functioning begins to occur and sets individuals on their own trajectories that have already been determined by late middle age/older adulthood (57,58). For example, using data from the National Survey of Health and Development, Murray et al. found that residents in neighborhoods with greater area deprivation at age 53 were associated with worse balance and chair-rise time, although, interestingly, no associations were observed with HGS (59). This finding suggests intervening in the neighborhood context earlier in the life course is needed for muscle health.

There are several strengths to this investigation. First, to the best of our knowledge, this is the first study examining the influence of both the built and social neighborhood environment on longitudinal trajectories of HGS, an important prognostic indicator of physical health (58). Second, we utilized nationally representative survey data. Therefore, our results are generalizable to community-dwelling adults aged 51 years and older in the United States. We also leveraged linked data from a novel neighborhood data repository that enabled us to undertake a multidimensional approach in considering both subjective and objective aspects of the built and social neighborhood environment (28). While prior work has examined the influence of neighborhoods on physical health and functioning (8,56), few studies have examined specific physiologic indicators like HGS. By focusing exclusively on HGS, our study offers an intriguing mechanistic picture of how and why neighborhoods may matter for physical functioning and disability. Despite these strengths, there are several limitations. First, we note that we adjusted for several variables that may be on the causal pathway between neighborhood disadvantage and HGS. Future work should undertake a formal mediation analysis to examine whether this relationship persists. Second, we incorporated subjective and objective measures of the neighborhood environment by aggregating information at multiple geographic levels, which may have introduced problems related to spatial scale, also known as the modifiable area unit problem. Third, we acknowledge the role of multiple testing in our model-building approach and, as a result, that some of our study findings may be due to statistical chance. Lastly, it is possible that selection bias may have been introduced into our analysis if sicker individuals may have moved to more disadvantaged neighborhoods.

The results from this investigation represent an important first step in aiding in our understanding of how neighborhood characteristics may influence HGS among middle-aged and older adults. This area of investigation is important because HGS, a reliable proxy of total body muscle strength, has been repeatedly shown to be associated with physical functioning and disability. Therefore, our study results provide initial evidence that focusing on individual neighborhood characteristics may hold promise in improving physical health and functioning. Our findings lay the groundwork for future research that seeks to uncover which specific features may potentially play the greatest role in HGS as a way of informing and designing interventions, particularly at the neighborhood level.

## Supplementary Material

glae242_suppl_Supplementary_Appendix

## References

[1] Yen IH , Fandel Flood J, Thompson H, Anderson LA, Wong G. How design of places promotes or inhibits mobility of older adults: realist synthesis of 20 years of research. J Aging Health. 2014;26(8):1340–1372. 10.1177/0898264314527610 24788714 PMC4535337

[2] Ratnayake M , Lukas S, Brathwaite S, Neave J, Henry H. Aging in place. Dela J Public Health. 2022;8(3):28–31. 10.32481/djph.2022.08.007 PMC9495472 36177171

[3] Simonsick EM , Guralnik JM, Volpato S, Balfour J, Fried LP. Just get out the door! importance of walking outside the home for maintaining mobility: findings from the women’s health and aging study. J Am Geriatr Soc. 2005;53(2):198–203. 10.1111/j.1532-5415.2005.53103.x 15673341

[4] Freedman VA , Grafova IB, Schoeni RF, Rogowski J. Neighborhoods and disability in later life. Soc Sci Med. 2008;66(11):2253–2267. 10.1016/j.socscimed.2008.01.013 18329148 PMC2478756

[5] Clarke P , Gallagher NA. Optimizing mobility in later life: the role of the urban built environment for older adults aging in place. J Urban Health. 2013;90(6):997–1009. 10.1007/s11524-013-9800-4 23592019 PMC3853178

[6] Rosso AL , Auchincloss AH, Michael YL. The urban built environment and mobility in older adults: a comprehensive review. J Aging Res. 2011;2011:1–10. 10.4061/2011/816106 PMC3134204 21766033

[7] Yu R , Wang D, Leung J, Lau K, Kwok T, Woo J. Is Neighborhood green space associated with less frailty? Evidence from the Mr. and Ms. Os (Hong Kong) Study. J Am Med Dir Assoc. 2018;19(6):528–534. 10.1016/j.jamda.2017.12.015 29402649

[8] Chaudhury H , Mahmood A, Michael YL, Campo M, Hay K. The influence of neighborhood residential density, physical and social environments on older adults’ physical activity: an exploratory study in two metropolitan areas. J. Aging Stud. 2012;26(1):35–43. 10.1016/j.jaging.2011.07.001 37841338 PMC10575573

[9] Beard JR , Blaney S, Cerda M, et al Neighborhood characteristics and disability in older adults. J Gerontol B Psychol Sci Soc Sci. 2009;64B(2):252–257. 10.1093/geronb/gbn018 PMC2655171 19181694

[10] Lang IA , Hubbard RE, Andrew MK, Llewellyn DJ, Melzer D, Rockwood K. Neighborhood deprivation, individual socioeconomic status, and frailty in older adults. J Am Geriatr Soc. 2009;57(10):1776–1780. 10.1111/j.1532-5415.2009.02480.x 19754500

[11] Latham K , Williams MM. Does neighborhood disorder predict recovery from mobility limitation? findings from the health and retirement study. J Aging Health. 2015;27(8):1415–1442. 10.1177/0898264315584328 25953811

[12] Duchowny KA , Clarke PJ, Peterson MD. Muscle weakness and physical disability in older Americans: longitudinal findings from the U.S. Health and Retirement Study. J Nutr Health Aging. 2018;22(4):501–507. 10.1007/s12603-017-0951-y 29582889 PMC6472265

[13] Gardner MP , Lightman S, Sayer AA, et al; Halcyon Study Team. Dysregulation of the hypothalamic pituitary adrenal (HPA) axis and physical performance at older ages: an individual participant meta-analysis. Psychoneuroendocrinology. 2013;38(1):40–49. 10.1016/j.psyneuen.2012.04.016 22658392 PMC3533133

[14] Peeters GMEE , Van Schoor NM, Visser M, et al Relationship between cortisol and physical performance in older persons. Clin Endocrinol (Oxf). 2007;67:398–406. 10.1111/j.1365-2265.2007.02900.x 17555515

[15] Peeters GMEE , van Schoor NM, van Rossum EFC, Visser M, Lips P. The relationship between cortisol, muscle mass and muscle strength in older persons and the role of genetic variations in the glucocorticoid receptor. Clin Endocrinol (Oxf). 2008;69(4):673–682. 10.1111/j.1365-2265.2008.03212.x 18248637

[16] Syddall H , Evandrou M, Cooper C, Aihie Sayer A. Social Inequalities in grip strength, physical function, and falls among community dwelling older men and women: findings from the Hertfordshire Cohort Study. J Aging Health. 2009;21(6):913–939. 10.1177/0898264309340793 19597159

[17] Juster FT , Suzman R. An overview of the Health and Retirement Study. J Hum Resour. 1995;30:S7–S56. 10.2307/146277

[18] Cheng FW , Gao X, Mitchell DC, et al Body mass index and all-cause mortality among older adults. Obesity (Silver Spring). 2016;24(10):2232–2239. 10.1002/oby.21612 27570944

[19] Bohannon RW. Muscle strength: clinical and prognostic value of hand-grip dynamometry. Curr Opin Clin Nutr Metab Care. 2015;18(5):465–470. 10.1097/MCO.0000000000000202 26147527

[20] Bohannon RW. Hand-grip dynamometry predicts future outcomes in aging adults. J Geriatr Phys Ther. 2008;31(1):3–10. 10.1519/00139143-200831010-00002 18489802

[21] Sayer AA , Kirkwood TBL. Grip strength and mortality: a biomarker of ageing? Lancet (London, England). 2015;386(9990):226–227. 10.1016/S0140-6736(14)62349-7 25982159

[22] Rantanen T , Harris T, Leveille SG, et al Muscle strength and body mass index as long-term predictors of mortality in initially healthy men. J Gerontol A Biol Sci Med Sci. 2000;55(3):M168–M173. 10.1093/gerona/55.3.m168 10795731

[23] Ortega FB , Silventoinen K, Tynelius P, Rasmussen F. Muscular strength in male adolescents and premature death: cohort study of one million participants. BMJ. 2012;345:e7279. 10.1136/bmj.e7279 23169869 PMC3502746

[24] Crimmins EM , Guyer H, Langa KM, Ofstedal MB, Wallace RB, Weir DR. Documentation of Physical Measures, Anthropometrics and Blood Pressure in the Health and Retirement Study. Survey Research Center, Institute for Social Research, University of Michigan; 2008.

[25] Landi F , Calvani R, Picca A, Marzetti E. Can Muscle Strength Be Considered a Composite Biomarker of Sarcopenia? The Measure of Handgrip Strength in Research and Daily Practice. JAMDA. 2018;19(5):373–374. 10.1016/j.jamda.2018.01.006 29559370

[26] Sallis JF , Cervero RB, Ascher W, Henderson KA, Kraft MK, Kerr J. An ecological approach to creating active living communities. Annu Rev Public Health. 2006;27:297–322. 10.1146/annurev.publhealth.27.021405.102100 16533119

[27] Clark WA , Coulter R. Who wants to move? The role of neighbourhood change. Environ Plan A. 2015;47(12):2683–2709. 10.1177/0308518x15615367

[28] National Neighborhood Data Archive (NaNDA). Inter-university Consortium for Political and Social Research (ICPSR). Accessed February 6, 2024. https://www.icpsr.umich.edu/web/ICPSR/series/1920

[29] Rollings KA , Dannenberg AL, Frumkin H, Jackson RJ. Built environment and public health: more than 20 years of progress. Am J Public Health. 2024;114(1):27–33. 10.2105/AJPH.2023.307451 38091569 PMC10726940

[30] Ailshire J , Melendez R, Chenoweth M. National Neighborhood Data Archive (NaNDA): Street Connectivity by Census Tract, United States, 2010. Published online 2021. 10.3886/E110641

[31] Clarke P , Melendez R, Chenoweth M. National Neighborhood Data Archive (NaNDA): Parks by Census Tract, United States, 2018. Published online 2020. 10.3886/E117921

[32] Jessica Finlay U of MI for SR, Mao Li U of MI for SR, Michael Esposito U of MI for SR, *et al*. National Neighborhood Data Archive (NaNDA): Grocery Stores by Census Tract, United States, 2003-2017. Published online October 1, 2020. 10.3886/E123001V1

[33] Philippa Clarke U of MI for SR, Robert Melendez U of MI for SR. National Neighborhood Data Archive (NaNDA): Public Transit Stops by Census Tract, United States, 2016-2018. Published online October 9, 2019. 10.3886/E111109V1

[34] Stephanie Miller U of M, Robert Melendez U of MI for SR, Megan Chenoweth U of MI for SR. National Neighborhood Data Archive (NaNDA): Urbanicity by Census Tract, United States, 2010. Published online January 11, 2021. 10.3886/E130542V1

[35] Jessica Finlay U of MI for SR, Mao Li U of MI for SR, Michael Esposito U of MI for SR. *et al*. National Neighborhood Data Archive (NaNDA): Arts, Entertainment, and Recreation Organizations by Census Tract, United States, 2003-2017. Published online October 26, 2020. 10.3886/E115543V2

[36] Yen IH , Kaplan GA. Neighborhood social environment and risk of death: multilevel evidence from the Alameda County study. Am J Epidemiol. 1999;149(10):898–907. 10.1093/oxfordjournals.aje.a009733 10342798

[37] Chen-Edinboro LP , Kaufmann CN, Augustinavicius JL, et al Neighborhood physical disorder, social cohesion, and insomnia: results from participants over age 50 in the Health and Retirement Study. Int Psychogeriatr. 2015;27(2):289–296. 10.1017/S1041610214001823 PMC4362806 25222023

[38] Melendez R , Clarke P, Khan A, Gomez-Lopez I, Li M, Chenoweth M. National Neighborhood Data Archive (NaNDA): Socioeconomic Status and Demographic Characteristics of Census Tracts, United States, 2008-2017. Published online 2020. 10.3886/E119451

[39] Michael Esposito U of MI for SR, Mao Li U of MI for SR, Jessica Finlay U of MI for SR, *et al*. National Neighborhood Data Archive (NaNDA): Law Enforcement Organizations by Census Tract, United States, 2003-2017. Published online October 20, 2020. 10.3886/E115973V3

[40] Crimmins EM , Kim JK, Langa KM, Weir DR. Assessment of cognition using surveys and neuropsychological assessment: the Health and Retirement Study and the Aging, Demographics, and Memory Study. J Gerontol B Psychol Sci Soc Sci. 2011;66B(suppl_1):i162–i171. 10.1093/geronb/gbr048 PMC3165454 21743047

[41] Lewinsohn PM , Seeley JR, Roberts RE, Allen NB. Center for Epidemiologic studies Depression scale (CES-D) as a screening instrument for depression among community-residing older adults. Psychol Aging. 1997;12(2):277–287. 10.1037//0882-7974.12.2.277 9189988

[42] Schisterman EF , Cole SR, Platt RW. Overadjustment bias and unnecessary adjustment in epidemiologic studies. Epidemiology (Cambridge, Mass). 2009;20(4):488–495. 10.1097/EDE.0b013e3181a819a1 19525685 PMC2744485

[43] Thompson CG , Kim RS, Aloe AM, Becker BJ. Extracting the variance inflation factor and other multicollinearity diagnostics from typical regression results. Basic and Applied Social Psychology. 2017;39(2):81–90. 10.1080/01973533.2016.1277529

[44] Germain CM , Vasquez E, Batsis JA, Mcquoid DR. Sex, Race and Age Differences in Muscle Strength and Limitations in Community Dwelling Older Adults: Data From the Health and Retirement Survey (HRS). Published online 2016. 10.1016/j.archger.2016.03.007 27017414

[45] Duchowny KA , Peterson MD, Clarke PJ. Cutpoints for clinical muscle weakness among older Americans. Am J Prev Med. 2016;53(1):63–69. 10.1016/J.AMEPRE.2016.12.022 PMC5497994 28190692

[46] Ross CE , Mirowsky J. Neighborhood disadvantage, disorder, and health. J Health Soc Behav. 2001;42(3):258–276. 10.2307/3090214 11668773

[47] Birnie K , Cooper R, Martin RM, et al; HALCyon study team. Childhood socioeconomic position and objectively measured physical capability levels in adulthood: a systematic review and meta-analysis. Vitzthum V, ed. PLoS One. 2011;6(1):e15564. 10.1371/journal.pone.0015564 21297868 PMC3027621

[48] Carney C , Benzeval M. Social patterning in grip strength and in its association with age; a cross sectional analysis using the UK Household Longitudinal Study (UKHLS). BMC Public Health. 2018;18:385. 10.1186/s12889-018-5316-x 29562880 PMC5863489

[49] Yusuf M , Montgomery G, Hamer M, McPhee J, Cooper R. Associations between childhood and adulthood socioeconomic position and grip strength at age 46 years: findings from the 1970 British Cohort Study. BMC Public Health. 2022;22:1427. 10.1186/s12889-022-13804-7 35883072 PMC9327373

[50] Fritz H , Cutchin MP, Gharib J, Haryadi N, Patel M, Patel N. Neighborhood characteristics and frailty: a scoping review. Gerontologist. 2020;60(4):e270–e285. 10.1093/geront/gnz072 31276582 PMC7768736

[51] Zare Sakhvidi MJ , Lafontaine A, Yang J, et al Association between outdoor air pollution exposure and handgrip strength: findings from the French CONSTANCES Study. Environ Health Perspect. 2022;130(5):57701. 10.1289/EHP10464 35559615 PMC9104871

[52] Lin H , Guo Y, Ruan Z, et al Association of indoor and outdoor air pollution with hand-grip strength among adults in six low- and middle-income countries. J Gerontol A Biol Sci Med Sci. 2020;75(2):340–347. 10.1093/gerona/glz038 30753311

[53] Labott BK , Bucht H, Morat M, Morat T, Donath L. Effects of exercise training on handgrip strength in older adults: a meta-analytical review. Gerontology. 2019;65(6):686–698. 10.1159/000501203 31499496

[54] Gedmantaite A , Celis-Morales CA, Ho F, Pell J, Ratkevicius A, Gray SR. Associations between diet and handgrip strength: a cross-sectional study from UK Biobank. Mech Ageing Dev. 2020;189:111269. 10.1016/j.mad.2020.111269 32479757

[55] Sandvik MK , Watne LO, Brugård A, Wang-Hansen MS, Kersten H. Association between psychotropic drug use and handgrip strength in older hospitalized patients. Eur Geriatr Med. 2021;12(6):1213–1220. 10.1007/s41999-021-00511-6 34033072 PMC8626357

[56] Lin BH , Ver Ploeg M, Kasteridis P, Yen ST. The roles of food prices and food access in determining food purchases of low-income households. J Pol Modeling. 2014;36(5):938–952. 10.1016/j.jpolmod.2014.07.002

[57] Cummins S , Flint E, Matthews SA. New neighborhood grocery store increased awareness of food access but did not alter dietary habits or obesity. Health Aff (Millwood). 2014;33(2):283–291. 10.1377/hlthaff.2013.0512 24493772 PMC4201352

[58] Duchowny KA , Ackley SF, Brenowitz WD, et al Associations between handgrip strength and dementia risk, cognition, and neuroimaging outcomes in the UK Biobank Cohort Study. JAMA Netw Open. 2022;5(6):e2218314. 10.1001/jamanetworkopen.2022.18314 35737388 PMC9227006

[59] Murray ET , Ben-Shlomo Y, Tilling K, Southall H, Aucott P, Kuh D, Hardy R. Area deprivation across the life course and physical capability in midlife: findings from the 1946 British Birth cohort. Am J Epidemiol. 2013;178(3):441–450. 10.1093/aje/kwt003 23788665 PMC3727343

