# Supplementary material for: The Neighborhood Environment and Handgrip Strength: Longitudinal Findings From the Health and Retirement Study
Source: J Gerontol A Biol Sci Med Sci. 2024 Oct 3;79(11):glae242. doi: 10.1093/gerona/glae242 (PMC11543991; doi:10.1093/gerona/glae242)
Supplement: glae242_suppl_Supplementary_Appendix [file glae242_suppl_supplementary_appendix.docx]

**Appendix**

*Sample Flow.* The figure below depicts how the analytic sample was determined.


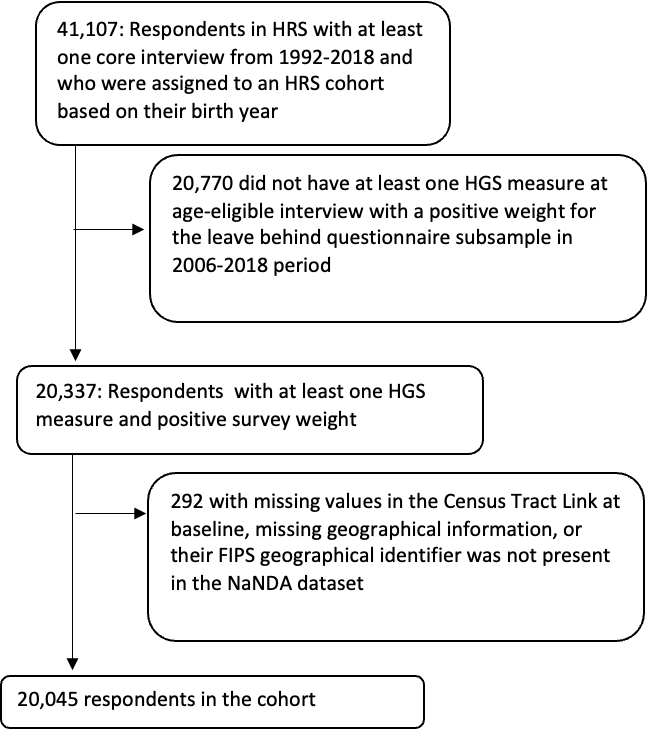


*Residential Moves.* From the table below, 15.74% of respondents with non-missing grip strength moved 1 or more times after baseline interview. Note that their move status was determined using only the TRACT10 variable based on guidance from the HRS website for all waves (excluding waves 1, 4, 7, 10, and 13), and geographic identifiers are based on where the interview was conducted. This location was not necessarily the respondent’s legal address. See complete HRS note:

*“For HRS Wave 1 (1992), AHEAD Wave 1(1993), and CODA/War Baby (1998), EBB (2004), MBB (2010) and LBB (2016), geographic identifiers reflect residence of respondents as recorded in the administrative database at baseline. For all other waves, geographic identifiers are based on the ISR administrative record of where the interview was conducted. This location was not necessarily the respondent’s legal address.”*

| **Number of changes in TRACT10 across core interviews after baseline** | | | | | |
| --- | --- | --- | --- | --- | --- |
| **tract10_change_postbasecount** | **Frequency** | **Unweighted Percent** | **Weighted Percent** | **Cumulative Frequency** | **Cumulative Percent** |
|  |  |  |  |  |  |
| **0** | 18737 | 84.23 | 84.26 | 18737 | 84.23 |
| **1** | 2999 | 13.48 | 13.33 | 21736 | 97.71 |
| **2** | 469 | 2.11 | 2.21 | 22205 | 99.82 |
| **3** | 40 | 0.18 | 0.20 | 22245 | 100 |

*Missing Data*. Based on the STATA manual, we followed guidance that states that the number of imputed datasets (M) should be >=100*(fraction of missing information-FMI) to provide an adequate level of reproducibility of MI analysis. Based on this information, we chose M = 30. From the multiple imputation estimates, the largest FMI = 0.2510 (males) / 0.0964 (females), so the number of imputations, 30, exceeds the required number. We also looked at the Relative Variance Increase (RVI). This statistic reports the increase in the variance of the estimate because of the loss of information about the parameter due to nonresponse relative to the variance of the estimate with no information lost. The closer this number is to 0, the less effect missing data have on the variance of the estimate. In our study, the average RVI is small: 0.0349 (males) / 0.0221 (females).

*Weights.* The rescaled weight was calculated as:

(wgtr/wgtr0)*(# of interviews with non-missing outcomes/respondent)/(sum of wgtr/wgtr0 for each respondent), where:

wgtr0: respondent weight for the leave behind questionnaire subsample at baseline interview (Tracker variable: xLBWGTR)

wgtr: respondent weight for the leave behind questionnaire subsample at each interview (Tracker variable: xLBWGTR)

*Model Checks and Assumptions.* The assumption of normality and linearity of HGS and the continuous covariates was assessed by visual examination using histogram with normal-density curve, quantile-quantile plots, and lowess plots of the observed outcome by the covariates. The outcome had a normal distribution. We log transformed population density and average block length, since their normality improved after transformation while also aiding in interpretation. We used a natural log transformation for covariates population density and average block length. We examined whether trajectories of handgrip strength change over time based on baseline exposure to the following neighborhood features: social cohesion, physical disorder, neighborhood disadvantage, number of transit stops, total area of open parks, population density, average block length, presence of gyms/fitness centers, street connectivity, law enforcement, and supermarkets. We used 3 restricted cubic splines for BMI with 4 knots estimated using Harrell’s recommended percentiles (21.1, 26.8, 31.1, and 40.9). The rest of continuous covariates in the model were used as linear terms. We checked for multicollinearity using the Variance Inflation Component (VIF) statistic.

*Collinearity.* In order to assess whether the neighborhood predictors were collinear with one another, we used the variance inflation factor (VIF). Generally, VIF values greater than 5 indicate multicollinearity may be present and values greater >10 indicate that multicollinearity is definitely an issue.^40^ Overall, VIF values were all under 5 with a mean VIF overall of 1.8, indicating low collinearity between predictors.

**
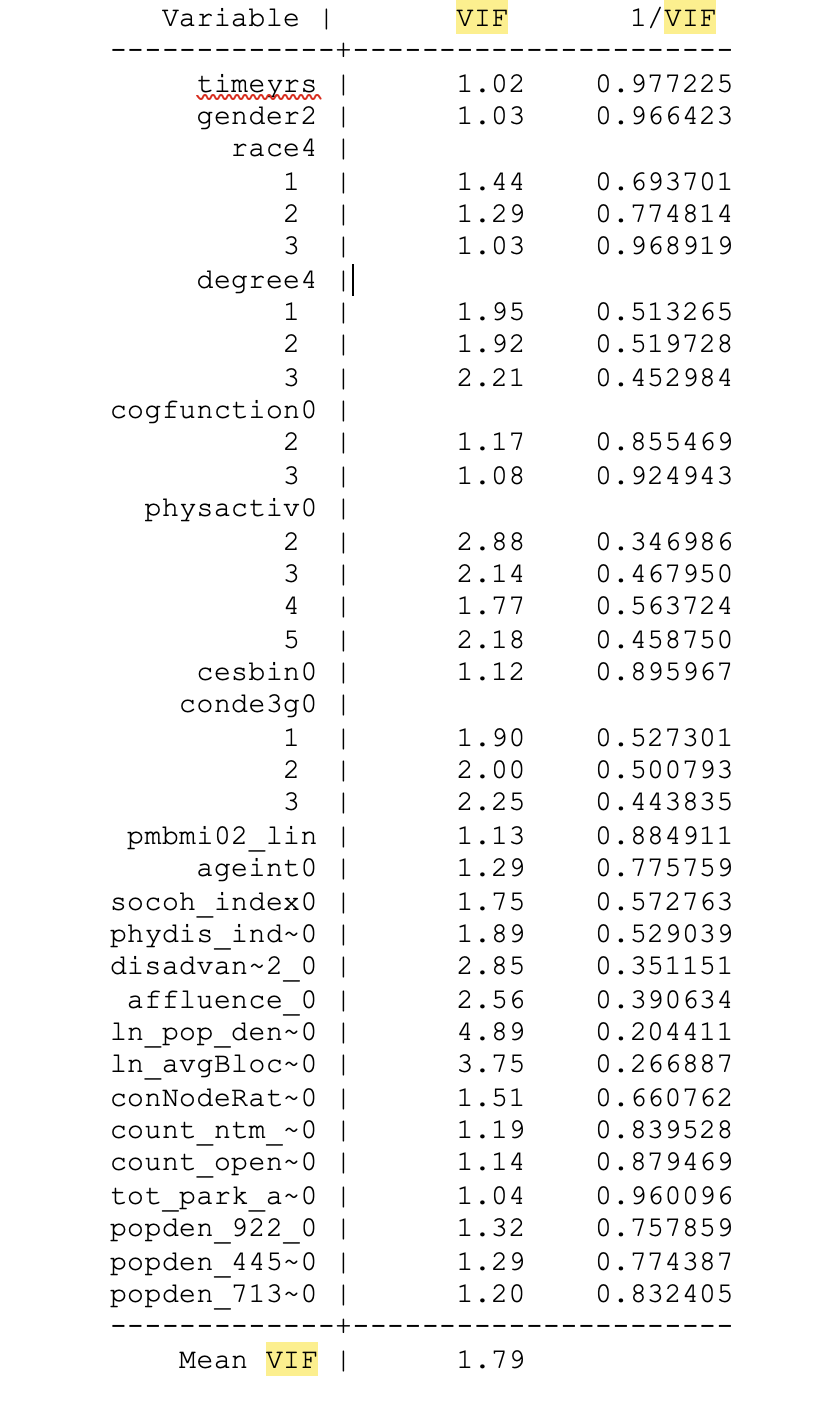
**

*Additional Sensitivity Analyses.* Since our longitudinal outcome variable HGS is correlated with respondent health status—and consequently the survival endpoint or the possibility of study dropout—as sensitivity analysis, we fit a joint unweighted model for longitudinal and survival data, obtaining maximum likelihood estimates via the expectation-maximization (EM) algorithm. We used SAS PROC NLMIXED as previously described.^15^ The use of the joint modeling did not meaningfully affect the results, so we chose the simpler model, the linear mixed effects model. We also assessed the effect of the survival endpoint or the possibility of study dropout using a mixed effects model with inverse probability weighting. These weights were computed as the inverse of the survival probability at each interview. We truncated the survival probabilities lower than the first percentile to the first percentile. The use of the mixed effects model with inverse probability weighting did not meaningfully affect the results, so we chose the simpler model, the linear mixed effects model.^16^
